# A machine learning-based prognostic predictor for stage III colon cancer

**DOI:** 10.1038/s41598-020-67178-0

**Published:** 2020-06-25

**Authors:** Dan Jiang, Junhua Liao, Haihan Duan, Qingbin Wu, Gemma Owen, Chang Shu, Liangyin Chen, Yanjun He, Ziqian Wu, Du He, Wenyan Zhang, Ziqiang Wang

**Affiliations:** 1 Department of Pathology, West China Hospital, Sichuan University, Chengdu, China; 2 Sichuan University-University of Oxford Huaxi Joint Centre for Gastrointestinal Cancer, West China Hospital, Sichuan University, Chengdu, China; 3 Department of Gastrointestinal Surgery, West China Hospital, Sichuan University, Chengdu, China; 4 College of Computer Science, Sichuan University, Chengdu, China; 5 The Institute for Industrial Internet Research, Sichuan University, Chengdu, China; 6 State Key Laboratory of Biotherapy and Cancer Center for Geriatrics, West China Hospital, Sichuan University, Chengdu, China; 7 Nuffield Division of Clinical Laboratory Sciences, Radcliffe Department of Medicine, University of Oxford, Oxford, United Kingdom; 8 West China College of Stomatology, Sichuan University, Chengdu, China

**Keywords:** Colon cancer, Machine learning, Tumour biomarkers

## Abstract

Limited biomarkers have been identified as prognostic predictors for stage III colon cancer. To combat this shortfall, we developed a computer-aided approach which combing convolutional neural network with machine classifier to predict the prognosis of stage III colon cancer from routinely haematoxylin and eosin (H&E) stained tissue slides. We trained the model by using 101 cancers from West China Hospital (WCH). The predictive effectivity of the model was validated by using 67 cancers from WCH and 47 cancers from The Cancer Genome Atlas Colon Adenocarcinoma database. The selected model (Gradient Boosting-Colon) provided a hazard ratio (HR) for high- *vs*. low-risk recurrence of 8.976 (95% confidence interval (CI), 2.824–28.528; *P*, 0.000), and 10.273 (95% CI, 2.177–48.472; *P*, 0.003) in the two test groups, from the multivariate Cox proportional hazards analysis. It gave a HR value of 10.687(95% CI, 2.908–39.272; *P*, 0.001) and 5.033 (95% CI,1.792–14.132; *P*, 0.002) for the poor *vs*. good prognosis groups. Gradient Boosting-Colon is an independent machine prognostic predictor which allows stratification of stage III colon cancer into high- and low-risk recurrence groups, and poor and good prognosis groups directly from the H&E tissue slides. Our findings could provide crucial information to aid treatment planning during stage III colon cancer.

## Introduction

Colorectal cancer (CRC) is one of the most common cancer diagnoses and is a leading cause of cancer-related deaths worldwide. In recent decades, validated predictive or prognostic biomarkers have facilitated chemotherapy or enabled targeted therapy selections for CRC. For example, different DNA mismatch repair/microsatellite instability (*MMR/MSI)* status combined with emergence of high-risk factors (lymphatic/vascular invasion, bowel obstruction, <12 lymph nodes harvested, and so on), different treatment strategies including observation, 5-fluorouracil chemotherapy alone, or combined chemotherapy, will be delivered to patients with stage II colon cancer^1^. For stage IV CRC, anti-*VEGF*, anti-*EGFR* (for *RAS*-wild type tumors), and even immunotherapy can be chosen based on *MMR/MSI* statuses^2^. Unfortunately, the gene statuses of *RAS, MMR/MSI*, and *BRAF*, which are used as predictive or prognostic markers in other stages of CRC, do not function as biomarkers to guide different treatment strategies in stage III colon cancer. Recent studies identified only T4 and/or N2 TNM staging to be eligible as a high-risk factor to instigate a longer duration of chemotherapy^3^. Finding a new prognostic predictor to stratify the patients to optimize therapy selection for stage III cancer is therefore an important issue.

Pathological tissue slides contain substantial amounts of information. In particular, this information can be exploited by the increasing employment of digital pathology and machine analysis techniques. Especially with the development of artificial intelligence (AI) in recent years, digital pathological based AI has become a crucial tool to solve many tough tasks. Some studies have reported that recurrence or survival prediction can be obtained directly from the H&E stained tissue slides using computer-aided systems in lung cancer^4^ and breast cancer^5^, and also outcome prediction in CRC^6,7^ using the tumor grade classification. However, the majority of studies utilized tissue microarrays (TMA) to perform research, which only contain a small portion of tumor area and may not well reflect the more complex real-world clinical practice. Furthermore, rarely do studies focus on stage III colon cancer.

Convolution neural networks (CNNs), a deep learning technique^8^, has revolutionized machine learning and developed into a mature frecognition technique which has been applied widely, i.e. facial recognition^9^, speech recognition^10^, document recognition^11^, and other aspects of image identification. In the medical AI field, CNNs have been utilized as major tools for the majority of image recognition studies. In radiation oncology, CNNs are used for lung cancer recognition based on CT images^12^, and auto-segmentation of CT images in many cancers^13^. In digital pathology, the studies mentioned above always chose CNNs as the primary tool.

In this study, we utilized CNNs and machine classifiers to develop a computer-aided predictor to stratify stage III colon cancers with high or low recurrent risk, and good or poor overall survival based on the H&E stained whole tissue slides. Furthermore, we validated the predictive power of the selected machine classifier by using histological images from the TCGA database to confirm its validity on application to tissue slides collected from other centers.

## Results

### Patient demographics and clinical characteristics

The clinicopathological features of this cohort of patients in our study were summarized in Supplementary Table 1. This group included 96 male and 72 female patients, and the median age was 61.5 years (range, 20–87 years). This group had 77 right colon cancers and 91 left colon cancers. For the primary tumor stage (T stage), it included one T1 case, eight T2 cancers, ninety-nine T3 cancers, and sixty T4 cancers. Follow-up time was from 1 to 122 months (mean 60 months, median 58 months). At the end of follow-up, 56 patients (33.3%) had tumor relapse or metastasis (range 1–100 months, mean 19.8 months), and 45 patients (26.8%) had died between 1–110 months (median 34 months).

### Machine classifier Gradient Boosting-Colon can predict the disease-free survival risk in stage III colon cancer

**Figure 1:**
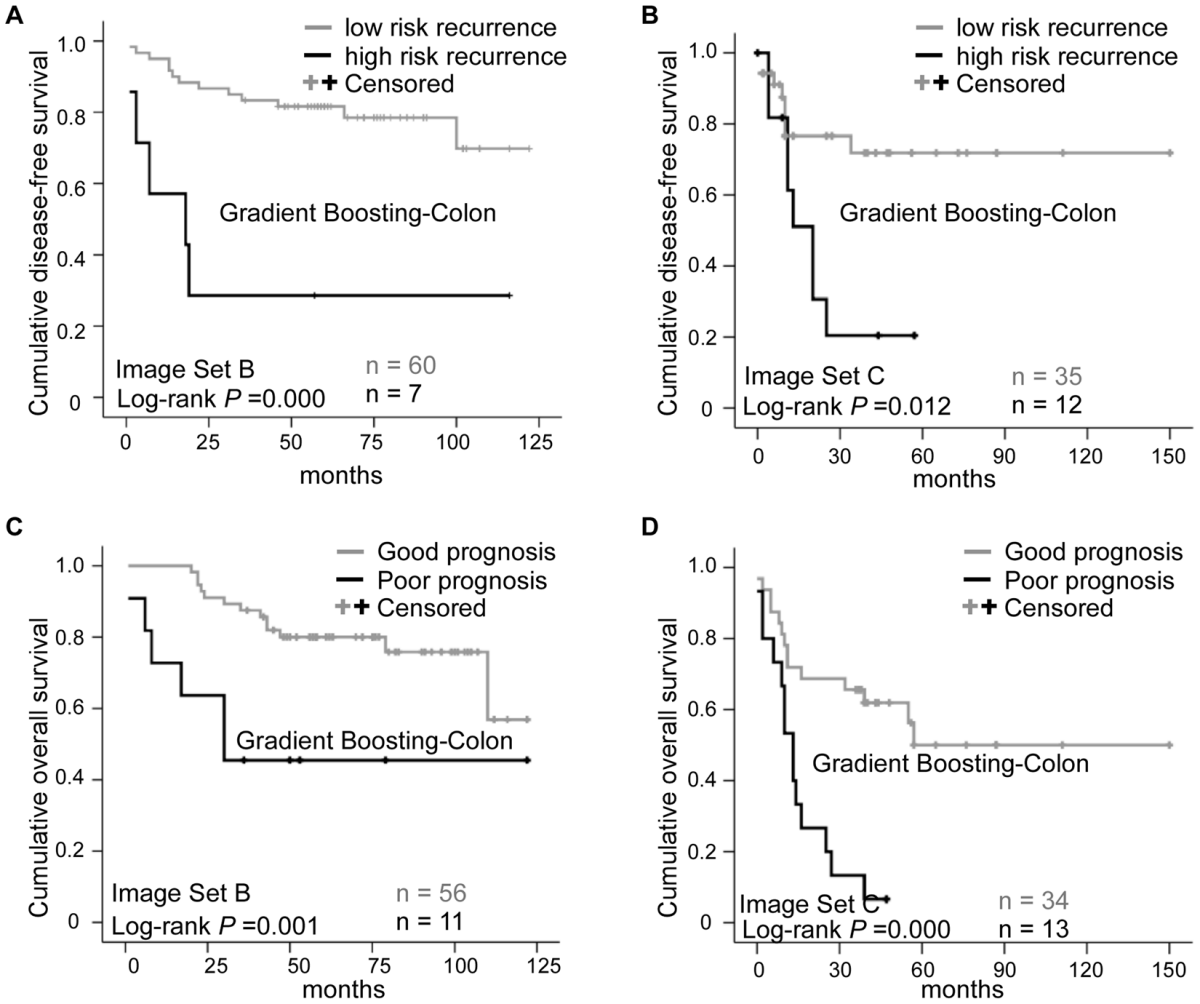
Prognostic prediction results for Image Set B and Image Set C. (**A**,**B**) represent the Kaplan-Meier plots for Gradient Boosting-Colon machine classifier using disease free survival as endpoint, (**C**,**D**) illustrate the Kaplan-Meier plots for Gradient Boosting-Colon machine classifier using overall survival as endpoint. (**A**,**C**) are the cases from Image Set B testing set, (**B**,**D**) are the cases from TCGA dataset. The number of cases in each category is indicated in the plots.

Kaplan-Meier survival curves showed that the Gradient Boosting-Colon classifier can correctly allocate the patients with stage III colon cancer into high-risk *vs* low-risk recurrence groups, with the *P* value of 0.000 and 0.012, in Image Set B and Image set C respectively (Fig. 1A,B). The result was confirmed in univariate (*P*, 0.002; HR, 5.397; 95%CI, 1.895–15.371) and multivariate (*P*, 0.000; HR, 8.976; 95%CI, 2.824–28.528) Cox regression analysis in Image Set B test group (Table 1). Similar results were obtained from Image Set C (univariate: *P*, 0.004; HR, 4.324; 95%CI, 1.588–11.775; multivariate: *P*, 0.003; HR, 10.273; 95%CI, 2.177–48.472.), which is summarized in Table 2. The average predictive accuracy in the whole test group was 75.5%.

**Table 1:** Univariate and multivariate Cox proportional hazards model based on disease-free survival (DFS) in Image Set B testing set.

Variable	Subtype	Univariate			Multivariate		
		*P*	HR	95%CI	*P*	HR	95%CI
AI DFS status	High- *vs*. low-risk	**0.002**	**5.397**	**1.895–15.371**	**0.000**	**8.976**	**2.824–28.528**
Age(y)	>50 *vs*.≤50	0.241	2.418	0.553–10.579	0.081	4.417	0.832–23.437
Gender	Male *vs*. Female	0.982	1.011	0.391–2.611	0.395	0.580	0.165–2.037
Tumor site	Left *vs*. Right colon	0.132	0.482	0.186–1.247	0.115	0.420	0.143–1.235
Tumor size	<5 cm *vs*. ≥5 cm	0.924	1.046	0.412–2.655	0.276	0.496	0.141–1.752
Histologic type	Muc + Sig *vs*. Ade	0.119	2.692	0.774–9.336	0.817	1.260	0.178–8.921
Histologic grade	G3 *vs*. G1 + G2	0.184	1.945	0.728–5.192	0.088	2.813	0.857–9.231
pT	T4 *vs*. T1–3	0.056	2.540	0.977–6.602	**0.036**	**3.080**	**1.079–8.792**
pN	N2 *vs*. N1	0.123	2.173	0.811–5.820	0.286	0.428	0.090–2.036
TNM stage	III C *vs*. IIIA + IIIB	**0.025**	**2.909**	**1.145–7.392**	**0.005**	**4.354**	**1.573–12.051**

Abbreviation: HR, hazard risk; CI, confidence interval; AI, artificial intelligence; Muc, mucous adenocarcinoma, Sig, signet ring cell adenocarcinoma; Ade, adenocarcinoma; G3, grade 3 (poor differentiation); G1, grade 1 (well differentiation); G2, grade 2 (moderated differentiation); pT, pathological primary tumor stage; pN, pathological lymph node stage; TNM, tumor, lymph node, and metastasis stage.

**Table 2:** Univariate and multivariate Cox proportional hazards model based on the disease-free survival (DFS) in the 47 patients from TCGA-COAD.

Variable	Subtype	DFS/Univariate			DFS/Multivariate		
		*P*	HR	95%CI	*P*	HR	95%CI
AI DFS status	High- *vs*. low-risk	**0.004**	**4.324**	**1.588–11.775**	**0.003**	**10.273**	**2.177–48.472**
Age(y)	>50 *vs*. ≤50	0.887	0.921	0.297–2.861	0.967	1.044	0.140–7.770
Gender	Male *vs*. Female	0.133	0.468	0.174–1.261	0.538	0.646	0.161–2.596
Tumor site	Left *vs*. Right colon	0.152	0.428	0.134–1.368	0.740	0.789	0.195–3.199
Histologic type	Muc + Sig *vs*. Ade	0.237	2.000	0.634–6.310	0.662	1.455	0.271–7.802
pT	T4 *vs*. T1–3	0.068	3.277	0.915–11.731	**0.033**	**4.731**	**1.130–19.809**
pN	N2 *vs*. N1	0.210	1.899	0.697–5.176	**0.041**	**10.316**	**1.102–96.585**
TNM stage	IIIC *vs*. IIIA + IIIB	0.716	1.215	0.425–3.478	0.054	0.108	0.011–1.042

Abbreviation: HR, hazard risk; CI, confidence interval; AI, artificial intelligence; Muc, mucous adenocarcinoma, Sig, signet ring cell adenocarcinoma; Ade, adenocarcinoma; pT, pathological primary tumor stage; pN, pathological lymph node stage; TNM, tumor, lymph node, and metastasis stage.

### Machine classifier Gradient Boosting-Colon can predict the overall survival risk in stage III colon cancer

The machine classifier Gradient Boosting-Colon was also an independent prognostic indicator used to stratify the patients into poor *vs* good prognosis groups (Log-rank *P*, 0.001 and 0.000 in Image Set B and C, respectively; Fig. 1C,D). Univariate cox proportional hazard model analysis showed the HR value of 5.047 (95% CI, 1.787–14.258; *P*, 0.002; Table 3) and 5.766 (95% CI, 2.475–13.433; *P*, 0.000; Table 4) in Image Set B and C respectively. Multivariate HR was 10.687 (95% CI, 2.908–39.272; *P*, 0.001; Table 3) in Image Set B test group, and 5.033 (95% CI, 1.792–14.132, *P*, 0.002; Table 4) in Image Set C. The average predictive accuracy in the whole test group was 77%.

**Table 3:** Univariate and multivariate Cox proportional hazards model based on overall survival (OS) in Image Set B testing set.

Variable	Subtype	Univariate			Multivariate		
		*P*	HR	95%CI	*P*	HR	95%CI
AI OS status	Poor *vs*. good	**0.002**	**5.047**	**1.787–14.258**	**0.001**	**10.687**	**2.908–39.272**
Age(y)	>50 *vs*. ≤50	0.209	3.673	0.483–27.937	0.233	3.693	0.431–31.662
Gender	Male *vs*. Female	0.665	1.256	0.447–3.533	0.756	1.209	0.365–4.002
Tumor site	Left *vs*. Right colon	0.351	0.611	0.217–1.719	0.110	0.416	0.141–1.222
Tumor size	<5 cm *vs*. ≥5 cm	0.921	1.052	0.382–2.903	0.847	0.898	0.299–2.699
Histologic type	Muc + Sig *vs*. Ade	0.253	2.092	0.590–7.417	0.856	0.862	0.174–4.274
Histologic grade	G3 vs. G1 + G2	0.060	3.004	0.956–9.437	0.206	2.443	0.613–9.739
pT	T4 *vs*. T1–3	**0.014**	**3.833**	**1.390–11.223**	**0.018**	**3.673**	**1.251–10.784**
pN	N2 *vs*. N1	0.131	2.218	0.789–6.237	0.377	0.476	0.092–2.470
TNM stage	IIIC *vs*. IIIA + IIIB	**0.017**	**3.466**	**1.253–9.589**	**0.022**	**3.306**	**1.189–9.191**

Abbreviation: HR, hazard risk; CI, confidence interval; AI, artificial intelligence; Muc, mucous adenocarcinoma, Sig, signet ring cell adenocarcinoma; Ade, adenocarcinoma; G3, grade 3 (poor differentiation); G1, grade 1 (well differentiation); G2, grade 2 (moderated differentiation); pT, pathological primary tumor stage; pN, pathological lymph node stage; TNM, tumor, lymph node, and metastasis stage.

**Table 4:** Univariate and multivariate Cox proportional hazards model based on the overall survival (OS) in the 47 patients from TCGA-COAD.

Variable	Subtype	OS/Univariate			OS/Multivariate		
		*P*	HR	95%CI	*P*	HR	95%CI
AI OS status	Poor *vs*. good	**0.000**	**5.766**	**2.475–13.433**	**0.002**	**5.033**	**1.792–14.132**
Age(y)	>50 *vs*. ≤50	0.110	2.661	0.801–8.836	0.183	2.982	0.598–14.878
Gender	Male *vs*. Female	0.569	0.806	0.382–1.697	0.608	1.296	0.481–3.492
Tumor site	Left *vs*. Right colon	0.219	0.597	0.262–1.359	1.516	1.414	0.496–4.029
Histologic type	Muc + Sig *vs*. Ade	0.306	1.613	0.645–4.034	0.238	2.075	0.618–6.968
pT	T4 *vs*. T1–3	**0.027**	**3.065**	**1.135–8.279**	**0.035**	**3.073**	**1.082–8.724**
pN	N2 *vs*. N1	**0.042**	**2.239**	**1.029–4.873**	**0.038**	**4.550**	**1.087–19.043**
TNM stage	IIIC *vs*. IIIA + IIIB	0.132	1.887	0.826–4.311	0.166	0.373	0.092–1.508

Abbreviation: HR, hazard risk; CI, confidence interval; AI, artificial intelligence; Muc, mucous adenocarcinoma, Sig, signet ring cell adenocarcinoma; Ade, adenocarcinoma; pT, pathological primary tumor stage; pN, pathological lymph node stage; TNM, tumor, lymph node, and metastasis stage.

### Identify morphologic parameters the Gradient Boosting-Colon potentially utilized

We analyzed the correlation between each morphologic parameter and the predictive recurrent risk from the whole test group (114 patients, Image set B and C), no significant was got (Table 5). In the survival prediction analysis, DEB_proportion (*P*, 0.012), TUM_median (*P*, 0.033), DEB_proportion /MUC_proportion (*P*, 0.005), DEB_proportion/STR_proportion (*P*, 0.031), DEB_ proportion/TUM_proportion (*P*, 0.005), DEB_mean (*P*, 0.025), DEB_median (*P*, 0.042), DEB/MUC_median (*P*, 0.013), DEB/STR_mean (*P*, 0.043), DEB/STR_median (*P*, 0.042), DEB/TUM_mean (*P*, 0.010), DEB/TUM_median (*P*, 0.042), and LYM/MUC_median (*P*, 0.042) significantly correlated (Kendall’s tab_b correlation coefficient: 0.2–0.4) with the predictive survival value (Table 5).

**Table 5:** *P* values of correlation test between the 45 morphological parameters with the predictive cancer recurrence risk and prognosis risk.

	Predicative recurrence risk			Predicative prognosis risk		
	ratio	mean	median	ratio	mean	median
DEB	0.735	0.886	0.854	**0.012**	0.025	0.042
LYM	0.753	0.568	0.430	0.284	0.711	0.137
MUC	0.274	0.654	0.430	0.135	0.128	0.092
STR	0.883	0.940	0.793	0.899	0.733	0.821
TUM	0.060	0.103	0.066	0.105	0.074	**0.033**
DEB/LYM	0.512	0.092	0.793	0.312	0.504	0.113
DEB/MUC	0.694	0.777	0.189	**0.005**	0.101	**0.013**
DEB/STR	0.952	0.149	0.854	**0.031**	**0.043**	**0.042**
DEB/TUM	0.264	0.338	0.189	**0.005**	**0.010**	**0.042**
LYM/MUC	0.142	0.314	0.189	0.630	0.078	**0.042**
LYM/STR	0.538	0.753	0.430	0.216	0.541	0.113
LYM/TUM	0.946	0.583	0.430	0.123	0.624	0.053
MUC/STR	0.467	0.301	0.733	0.357	0.307	0.497
MUC/TUM	0.163	0.205	0.066	0.540	0.884	0.258
STR/TUM	0.512	0.265	0.386	0.750	0.328	0.497

Abbreviation: DEB, debris; LYM, lymphocyte; MUC, mucus; STR, stroma; TUM, tumor.

## Discussion

One of the important demands for clinicians is to stratify patients who require different treatment strategies based on different prognoses, especially in the age of personalized medicine. However, for stage III colon cancer, the guidelines limit adjuvant chemotherapy to using fluoropyrimidines and/or oxaliplatin for 3 or 6 months. Furthermore, the survival of patients receiving 3 months adjuvant chemotherapy may be suboptimal compared to the 6 months, as only patients with N2 or/and T4 benefit from the 6-month duration of treatment^3^.

To develop new markers to guide treatment decisions or to predict the prognosis for the stage III colon cancer, we constructed a prognostic machine classifier, Gradient Boosting-Colon, for predicting patients DFS and OS, based on the digitized HE-stained whole slide images using a deep learning framework. We confirmed the predictive power of this machine classifier in two different datasets, both with accurate performance. Thus, we present a novel prognostic predictor which can be integrated into the treatment discussion in the future clinical workflow.

Prognostic prediction using a digital image-based computer system, is an economic and time saving approach, which prevents additional tissue destruction and could increase objectivity. A growing number of laboratories are digitalizing, leading to a new trend of gradually increasing application of some standardized computer modules to facilitate the daily clinical practice.

Prognostic predictors generated from artificial intelligence techniques in CRC was reported in two studies^6,7^. Both the studies focus on the all stages of CRC, which compare the predicative ability between deep learning technique and the current tumor staging system, also the predicative power between the new technique and the human pathologist, or even compared with some genetic biomarkers. However, this present study is the first study specifically trying to stratify the patients of stage III colon cancer into high or low recurrent risk groups, moreover, into good and poor prognosis, which might provide evidence to help treatment decision making. Furthermore, the high risk and low risk recurrent groups classified by Gradient Boosting-Colon classifier differed about 4–5 times in HR in univariable analysis and about 8–10 times in multivariable analysis in the individual two test sets, which the HR value was higher than that of the T stage, N stage (similar) and TNM stage. For the good or poor overall survival analysis, the poor and good overall survival groups assigned by Gradient Boosting-Colon classifier differed about 5 times in HR in univariable analysis and about 5–10 times in multivariable analysis, which the HR value was higher than the T stage, N stage and TNM stage. It might be reasonable to believe that the patients with high-risk recurrence or poor prognosis estimated by using Gradient Boosting-Colon classifier would receive a longer duration of treatment, or even enrolled into specific clinical trials to access more aggressive treatment strategies.

We are trying to unveil the morphologic parameters Gradient Boosting-Colon classifier potentially utilized. Interestingly, the parameters related to tumor necrosis (DEB) significantly correlated with the predictive survival risk, which gave a hint that the tumor necrosis is an important morphologic indicator. The parameter of lymphocyte/mucous_median also correlated with the survival prediction, which was consistent with the concept that the immune micromovement is curial for cancer treatment response and patient prognosis^14^. However, the statistically significant parameters only moderately correlated with the predictive survival risk, and nothing was got for the cancer recurrence risk analysis. Combinations of parameters with more complexity might be needed for further analysis, as only 45 morphologic parameters were included in this study.

The strengths of the present study include the generation of a new biomarker for stage III colon cancer, which has rare validated predictive or prognostic marker currently. Secondly, using digital images of routine H&E tissue section provides a cost-effective and time-saving approach, compared to genetic testing which we currently utilize to guide treatment decisions in clinical practice. Thirdly, the automated analysis procedure can reduce human intervention, and increase objectivity and reproducibility.

Our study did have some limits. Just as all the studies employing deep learning methods, the question is which features the machine utilized, and what the machine classifier exactly represents. The CNN quantifiers the different components of the whole slides, a machine classifier re-weights the different components by using the existing prognosis data, to get a predictive classifier, which is not easily completed by pathologists. Another limitation was the relatively small sample size used. We utilized the H&E images from the TCGA database to the remedy this defect, although there is only a small cohort of stage III colon cancer cases with histological images available from the current public datasets. However, applying the TCGA cases can confirm the predictive power of our machine classifier, and can illustrate that this machine classifier can be applied to H&E staining images made by various H&E staining machines, or for patients of different races, and other H&E variations. Further work is needed to confirm this machine classifier by using larger numbers of cases in order to promote direct translation to the clinic.

In summary, we employ a CNN model and a machine classifier to construct an independent predictive marker, based on digital H&E whole slide images in a cohort of 168 stage III colon cancer patients from our institution, and a cohort of 47 patients from the TCGA database. The stratification of stage III colon cancer patients into low- or high-risk recurrence, and good or poor survival groups provides prognostic significance which could aid treatment planning. We believe this is a critical first step to use this kind of economic, non-tissue destructive, and result readily available computer method to develop a predictive classifier to stratify stage III colon cancers. However, a larger validation dataset is needed to further confirm this classifier in order to reach clinical standards in the near future.

## Materials and Methods

### Patients and treatment

This study was approved by the West China Hospital Institutional Review Board. From December 2008 to December 2015, 210 patients with stage III colon adenocarcinoma treated with curative resection and followed by FOLFOX or CAPOX chemotherapy (3 or 6-months duration) at our institution were collected for this retrospective study. 177 patients with complete follow-up data were collected, with a follow-up rate of 84.3%. We excluded 9 patients due to non-cancer related deaths such as heart and lung failure, amounting to a final total of 168 patients enrolled in this study. The patient selection procedure is presented in Supplementary Fig. 1. All patients had tissue slides of surgical specimens. TNM stage was reviewed following the American Joint Committee on Cancer (AJCC) 8^th^ edition of cancer staging system.

Disease-free survival (DFS) was calculated from initial diagnosis to the first event (local recurrence/progression, distant recurrence, or disease-related death). The overall survival (OS) was calculated from initial diagnosis to death from disease-related death, or the last date of follow-up. The follow-up time was from 1 to 122 months (mean 60 months, median 58 months). Based on the previous clinical trial set up 3-year DFS as the endpoint^3^, we chose 3-year (36-monthe) as the cut-off value for our analysis. For the DFS analysis, patients were divided into two groups corresponding to those with tumor relapse or metastasis after treatment within 36 months (high-risk recurrence), and those without tumor relapse within 36 months (low-risk recurrence). For the OS prediction, patients dying of cancer-related disease within 36 months were defined as the poor prognosis group, and patients surviving without tumors within 36 months were defined as the good prognosis group.

### Images data set

Three H&E-stained image sets were used in this study. All the images were 0.5μm/px, and the normalization method of dividing each pixel by 255 was adopted.

Image Set A: a public H&E-stained image dataset of colorectal cancer (https://zenodo.org/record/1214456#.XhsdpTNKg54) comprised of 100,000 image patches each with a resolution of 224 × 224 pixels (px). These images were annotated with nine categories: background (BACK), adipose tissue (ADI), debris (DEB), lymphocytes (LYM), mucus (MUC), smooth muscle (MUS), normal colon mucosa (NORM), stroma (STR), and colorectal adenocarcinoma (TUM). The data set was utilized to train the CNNs to identify the category of each image patch.

Image Set B: an image set of 168 whole tissue slides from the 168 surgical specimens in West China Hospital, which was used for the modeling and testing of the automated computer-aided predictor. The images were scanned by using the NanoZoomer2.0-RS scanner (Hamamatsu Photonics, Japan), which have resolution from 40960 × 41472 to 135168 × 107008. Cases were randomly assigned into two sets: 101 cases as modeling set for training the classifier, and 67 cases as a test set for independent validation (Supplemental Tables 2 and 3).

Image Set C: a public dataset of fifty-four stage III colon cancers with more than 36 months follow-up from TCGA-COAD (https://portal.gdc.cancer.gov/projects/TCGA-COAD) were retrieved. Cases with image sizes less than 50 kb were excluded due to being unclear when magnified, resulting in 47 cases with tissue slide images (Supplemental Table 4). This collection was used as multicenter data to further validate the effectiveness of the selected machine classifier.

### Model training and classifier construction

**Figure 2:**
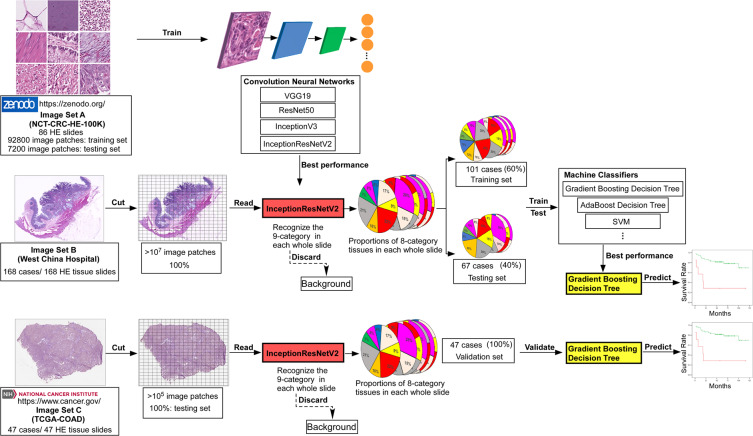
Flowchart of this study. Briefly, Image Set A (image patches which were annotated as 9-categry in tissue slides from colorectal cancer, downloaded from the published database) was used as training set to train multiple neural networks (CNNs). The InceptionResNet V2 was locked-down after category-recognition training, due to highest accuracy in to recognizing the image patches from Image Set B and calculating the proportions of each tissue category in each whole slide (pie charts), after discarding Background. Image Set B was separated into training set (60%) and test set (40%), and the training set with the proportions of 8-tissue category was sent into multiple machine classifiers to construct the predictive model. The test set was applied to test the accuracy of each machine predictive model. Validated the performance of each predictive model by using Image Set C. Finally, Gradient Boosting Decision Tree was chosen as our predictive model.

**Figure 3:**
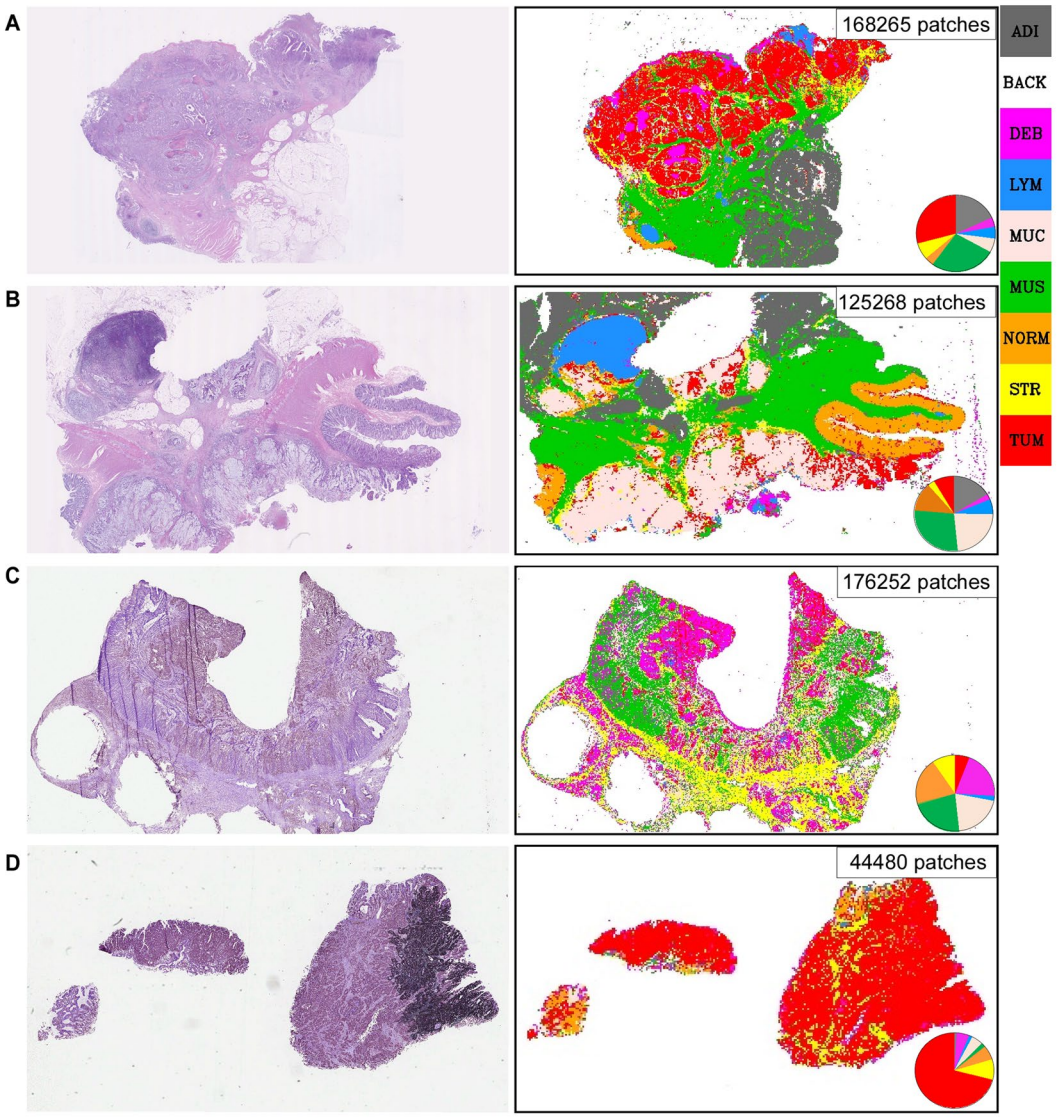
A neural network (CNN) segmented and restored the H&E whole tissue slides. The CNN, InceptionResNetV2 was used to recognize the nine categories (ADI, adipose tissue; BACK, background; DEB, debris; LYM, lymphocyte; MUC, mucus; MUS, muscle; NORM, normal mucosa; STR, stroma; TUM, tumor) in each whole tissue slides from the Image Set B and C. Left panel showed the original H&E staining tissue slides, the right panel was the classification maps restored by CNN, the pie charts showed the proportions of each tissue category. (**A**) typical adenocarcinoma and (**B**) mucous adenocarcinoma were from the Image Set B. (**C**,**D**) were from the Image Set C. (**C**) showed some problems caused by handcraft, such as tissue fold and hollowing, (**D**) presented visualization problems caused by uneven fixation and covering of the slides. Despite these imperfections in the whole tissue slides, the trained CNN still can perfectly recognize the different tissue categories.

The flow chart illustrating the procedure of training and constructing the machine model is presented in Fig. 2, and the machine auto-identification of the whole slide images are shown in Fig. 3.

Convolutional neural networks (CNN) are a kind of Feedforward Neural Networks that contain convolution computation and have depth structure^15^. It is one of the representative algorithms of deep learning and has been gradually used in medical research^16^. Image Set A was applied to train the CNNs to recognize the different categories of tissue patches in the whole slides. We randomly chose 800 image patches from each category, 7,200 in total, as a test set, and the residual 92,800 images patches were assigned to the training set. Several CNNs (VGG19^17^, ResNet50^18^, InceptionV3^19^, InceptionResNetV2^20^), which were pre-trained on the ImageNet database (www. image-net.org), were trained and tested using these training and test sets. Finally, we chose InceptionResNetV2 to carry out further experiments, due to achieving the best performance accuracy of 99%. The identification accuracy of each CNN is summarized in Supplementary Table 5.

After being trained by Image Set A, the selected InceptionResNetV2 model, which had the ability to recognize different (nine-category) components from whole tissue slides of colorectal adenocarcinoma, was applied to recognize the images patches of Image Set B. The whole-slide images were cut as patches with resolution of 224 × 224 (one whole-slide can be cut into 100,000–300,000 image patches), and pass through the InceptionResNetV2 model to recognize the categories of each patch. We adopt the Adaptive Moment Estimation (Adam) [1] optimizer with the initial learning rate of 0.00001. The proportions of each tissue category (eight-categories) in each whole-slide were counted, after BACK was dismissed. The proportions of each tissue category were employed as features for the prediction of recurrence and outcome in stage III colon cancer.

Constructing the DFS prediction involved randomly dividing the cases of Image Set B five times into the training set and test set, with a 6:4 ratio. The same method was used for separation of training and test groups for OS analysis. No significant differences in the major clinicopathological features between each training and testing group were detected (Supplementary Tables 2 and 3).

Next, we trained nine machine classifiers on each slide (with eight-category proportions) of the training set, and the predictive power was tested on each test set (Supplementary Table 6). Finally, the Gradient Boosting Decision Tree machine classifier showed the best performance, when using five-fold cross-validation and Jackknife test^21^ within these test sets. Thus, the Gradient Boosting Decision Tree classifier (Supplementary explanation) was locked down, further named as Gradient Boosting-Colon, to be validated on another test set. The classifiers used in this article all the application programming interface provided by the python package scikit-learn^22^.

Forty-seven cases of stage III colon cancers were retrieved from Image Set C, where the clinical data (Supplementary Table 4) and tissue slide images (Image Set C) of these cases were used as multicenter data to further validate the effectiveness of the selected machine classifier.

CNNs and machine classifiers training and testing was done in Python on two standard desktop workstations with 4 kernel processors (Intel Core i7 7700 @ 3.6 Hz) and an NVIDIA GeForce 1080Ti(11GB) with 168 GB RAM.

### Morphologic parameters

We recalculated the proportions of five tissue categories (DEB, LYM, MUC, STR, and TUM), after discarding the normal components (ADI, MUS, and NORM). To analysis more parameters the machine classifier might utilize, we generated new parameters by combing each two tissue categories, such as DEB_proportion) /LYM_proportion, DEB_proportion)/MUC _proportion etc., which got 15 continuous variable parameters (5 original proportion, and 10 combined ratios). Each case was assigned to <mean or >mean group, and <median or >median group, by applying mean value and median value as cutoff value to generate new parameters (categorical variable parameters). Finally, 45 morphologic parameters were got (Table 5).

### Statistical analysis

The survival analysis was performed on the test set (Image Set B and C) only. Each case (each image) was assigned a dichotomous possibility (either high- or low-risk) of tumor recurrence, and possibility of outcome (either good or poor), using different machines classifiers. A comparison between the predicted labels and actual follow-up outcome was performed for each machine classifier to estimate the performance of the classifiers. Estimated risk stratification possibilities were illustrated by using the Kaplan-Meier method and the differences were compared using the Log-rank test^23^. Hazard ratios were evaluated using the univariate and multivariate Cox proportional hazards model^24^. The differences between each major clinicopathological feature and prognosis were analyzed by using univariate and multivariate Cox proportional hazards model. The difference of the major clinicopathological features between the training group and test group was analyzed by Pearson Chi-Square test. The correlation between the morphologic parameters and the dichotomous predictive risk generated by machine classifier was analyzed, whose significance was tested by Kendall’s tab_b Correlation (continuous variable) and Pearson Chi-square (categorical variable).

Analyses were performed using SPSS 23.0 software. Two-sided p < 0.05 was considered statistically significant.

### Other statements

All methods were carried out in accordance with relevant guidelines and regulations. Informed consent was obtained from all subjects or, if subjects are under 18, from a parent and /or legal guardian.

## Supplementary information

Supplementary information.

## References

[1] Benson AR (2004). American Society of Clinical Oncology recommendations on adjuvant chemotherapy for stage II colon cancer. J Clin Oncol.

[2] NCCN colon carcinoma treatment guidelines, https://www.nccn.org/default.aspx.

[3] Grothey A (2018). Duration of Adjuvant Chemotherapy for Stage III Colon Cancer. N Engl J Med.

[4] Corredor G (2019). Spatial Architecture and Arrangement of Tumor-Infiltrating Lymphocytes for Predicting Likelihood of Recurrence in Early-Stage Non-Small Cell Lung Cancer. CLIN CANCER RES.

[5] Lu C (2018). Nuclear shape and orientation features from H&E images predict survival in early-stage estrogen receptor-positive breast cancers. LAB INVEST.

[6] Kather JN (2019). Predicting survival from colorectal cancer histology slides using deep learning: A retrospective multicenter study. PLOS MED.

[7] Bychkov D (2018). Deep learning based tissue analysis predicts outcome in colorectal cancer. Sci Rep.

[8] LeCun Y, Bengio Y, Hinton G (2015). Deep learning. NATURE.

[9] Parkhi, O. M., Vedaldi, A. & Zisserman, A. Deep face recognition. *BMVC*, Vol. 16 (2015).

[10] Dahl GE (2011). Context-Dependent Pre-Trained Deep Neural Networks for Large-Vocabulary Speech Recognition. IEEE Transactions on Audio, Speech, and Language Processing.

[11] D., S.M., U., B. & S., K.P. CNN based common approach to handwritten character recognition of multiple scripts. *13th International Conference on Document Analysis and Recognition (ICDAR)*, 1021–1025 (2015).

[12] Sun, W., Zheng, B. & Qian, W. Computer aided lung cancer diagnosis with deep learning algorithms. *In Medical imaging 2016: computer-aided diagnosis*, Vol. 9785 97850Z (International Society for Optics and Photonics, 2016).

[13] Ibragimov B, Xing L (2017). Segmentation of organs-at-risks in head and neck CT images using convolutional neural networks. MED PHYS.

[14] Galon J, Bruni D (2019). Approaches to treat immune hot, altered and cold tumours with combination immunotherapies. NAT REV DRUG DISCOV.

[15] Schmidhuber J (2015). Deep learning in neural networks: An overview. Neural Networks.

[16] Ta, N., Li, H., Liu, S. & Zuo, Y. Mining Key Regulators of Cell Reprogramming and Prediction Research Based on Deep Learning Neural Networks. *IEEE ACCESS* **PP**, 1 (2020).

[17] Simonyan, K. & Zisserman, A. Very deep convolutional networks for large-scale image recognition. *arXiv preprint arXiv:1409.1556* (2014).

[18] He, K., Zhang, X., Ren, S. & Sun, J. Deep residual learning for image recognition. *Proceedings of the IEEE conference on computer vision and pattern recognition*, 770–778 (2016).

[19] Szegedy, C., Vanhoucke, V., Ioffe, S., Shlens, J. & Wojna, Z. Rethinking the inception architecture for computer vision. *Proceedings of the IEEE conference on computer vision and pattern recognition*, 2818–2826 (2016).

[20] Szegedy, C., Ioffe, S., Vanhoucke, V. & Alemi, A.A. Inception-v4, inception-resnet and the impact of residual connections on learning. *Thirty-first AAAI conference on artificial intelligence*. (2017).

[21] Shao, J. & Tu, D. *The jackknife and bootstrap*. (Springer Science & Business Media, 2012).

[22] Pedregosa F (2011). Scikit-learn: Machine learning in Python. J MACH LEARN RES.

[23] Goel MK, Khanna P, Kishore J (2010). Understanding survival analysis: Kaplan-Meier estimate. International journal of Ayurveda research.

[24] Bender R, Augustin T, Blettner M (2005). Generating survival times to simulate Cox proportional hazards models. STAT MED.

