# Supplementary material for: A machine learning-based prognostic predictor for stage III colon cancer
Source: Sci Rep. 2020 Jun 25;10:10333. doi: 10.1038/s41598-020-67178-0 (PMC7316723; doi:10.1038/s41598-020-67178-0)
Supplement: Supplementary file 1 — Supplementary information. [file 41598_2020_67178_MOESM1_ESM.pdf]

A machine learning-based prognostic predictor for stage III colon cancer

Running title: Using H&E stained whole tissue slides to inform treatment decisions

Dan Jiang[1,2], Junhua Liao[4,5], Haihan Duan[4,5], Qingbin Wu[3,6], Gemma Owen[7], Chang Shu[8], Liangyin Chen[4,5], Yanjun He[1], Ziqian Wu[8], Du He[1,2], Wenyan Zhang[1,2*], Ziqiang Wang[2,3 *]

Authors' Affiliations:

1 Department of Pathology, West China Hospital, Sichuan University, Chengdu, China.

2 Sichuan University-Oxford University Huaxi Gastrointestinal Cancer Center, West China Hospital, Sichuan University, Chengdu, China.

3 Department of Gastrointestinal Surgery, West China Hospital, Sichuan University, Chengdu, China.

4 College of Computer Science, Sichuan University.

5 The Institute for Industrial Internet Research, Sichuan University.

6 State Key Laboratory of Biotherapy and Cancer Center for Geriatrics, West China Hospital, Sichuan University, Chengdu, China.

7 Nuffield Division of Clinical Laboratory Sciences, Radcliffe Department of Medicine, University of Oxford, Oxford, United Kingdom.

8 West China College of Stomatology, Sichuan University, Chengdu, China.

Dan Jiang and Junhua Liao contributed equally to this work.

[*] **Correspondence to:** Wenyan Zhang, Department of Pathology, West China Hospital, Sichuan University, No.37 Guoxue Alley, Chengdu, Sichuan 610041, China; E-mail: zhangwenyanpath@163.com.

Ziqiang Wang, Department of Gastrointestinal Surgery, West China Hospital, Sichuan University, No.37 Guoxue Alley, Chengdu, Sichuan 610041, China. E-mail: wangziqiang@scu.edu.cn.

Supplementary material

Supplementary Figure 1. Patients selection of the Image Set B.

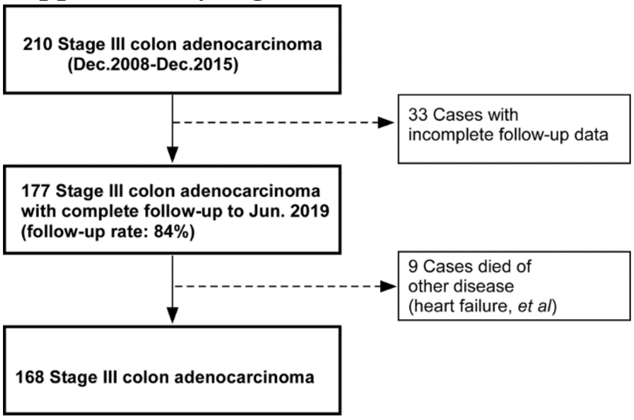

Supplementary Table 1 Clinical pathological features of the Image set B of 168 patients with stage III colon cancer.

Variable	Subtype	n	%
Age(y)	≤50	35	20.8
	>50	133	79.2
Gender	Male	96	57.1
	Female	72	42.9
Tumor site	Right colon	77	45.8
	Left colon	91	54.2
Tumor size	<5 cm	92	54.8
	≥5 cm	76	45.2
Histologic type	Adenocarcinoma	148	88.1
	Mucinous adenocarcinoma	18	10.7
	Signet ring cell adenocarcinoma	2	1.2
Histologic grade	G1+G2	86	51.2
	G3	82	48.8
pT stage	T1	1	0.6
	T2	8	4.8
	T3	99	58.9
	T4	60	35.9
pN stage	N1a	69	41.1
	N1b	36	21.4
	N1c	19	11.3
	N2a	27	16.1
	N2b	17	10.1
TNM stage	IIIA	10	6
	IIIB	105	62.5
	IIIC	53	31.5

Abbreviation: G1, grade 1 (well differentiation); G2, grade 2 (moderated differentiation); G3, grade 3 (poor differentiation); pT, pathological primary tumor stage; pN, pathological lymph node stage; TNM, pathological tumor, lymph node, and metastasis stage.

Supplementary Table 2 Clinicopathological features of DFS training set and test set of this cohort of 168 patients.

		Training set		Test set		*P*
Variable	Subtype	n=101	%	n=67	%	
Age(y)	≤50	21	20.8	14	20.9	0.987
	>50	80	79.2	53	79.1	
Gender	Male	56	55.4	40	59.7	0.588
	Female	45	44.6	27	40.3	
Tumor site	Right colon	47	46.5	30	44.8	0.824
	Left colon	54	53.5	37	55.2	
Tumor size	<5 cm	54	53.5	38	56.7	0.681
	≥5 cm	47	46.5	29	43.3	
Histologic type	G1+G2	56	55.4	30	44.8	0.599
	G3+special type	45	44.6	37	55.2	
pT	T1+T2	8	7.9	1	1.5	0.645
	T3	56	55.4	43	64.2	
	T4	37	36.6	23	34.3	
pN	N1	72	71.3	53	79.1	0.258
	N2	29	28.7	14	20.9	
TNM	IIIA	8	7.9	2	3	0.278
	IIIB	63	62.4	42	62.7	
	IIIC	30	29.7	23	34.3	
DFS status	Non-recurrence	63	62.4	49	73.1	0.149
	Recurrence	38	37.6	18	26.9	
OS status	Alive	71	70.3	52	77.6	0.297
	Death	30	29.7	15	22.4	

Abbreviation: G1, grade 1 (well differentiation); G2, grade 2 (moderated differentiation); G3, grade 3 (poor differentiation). Special type: includes mucinous adenocarcinoma and signet ring cell adenocarcinoma; pT, pathological primary tumor stage; pN, pathological lymph node stage; TNM, tumor, lymph node, and metastasis stage.

Supplementary Table 3 Clinicopathological features of OS training set and test set of this cohort of 168 patients.

		Training set		Test set		*P*
Variable	Subtype	n=101	%	n=67	%	
Age(y)	≤50	22	21.8	13	19.4	0.712
	>50	79	78.2	54	80.6	
Gender	Male	54	53.5	42	62.7	0.24
	Female	47	46.5	25	37.3	
Tumor site	Right colon	44	43.6	33	49.3	0.472
	Left colon	57	56.4	34	50.7	
Tumor size	<5 cm	60	59.4	32	47.8	0.139
	≥5 cm	41	40.6	35	52.2	
Histologic type	G1+G2	53	52.5	33	49.3	0.765
	G3+special type	48	47.5	34	50.7	
pT	T1+T2	6	5.9	3	4.5	0.855
	T3	59	58.4	40	59.7	
	T4	36	35.6	24	35.8	
pN	N1	75	74.3	50	74.6	0.957
	N2	26	25.7	17	25.4	
TNM stage	IIIA	7	6.9	3	4.5	0.747
	IIIB	60	59.4	45	67.2	
	IIIC	34	33.7	19	28.4	
DFS status	Non-recurrence.	68	67.3	44	65.7	0.825
	Recurrence	33	32.7	23	34.3	
OS status	Alive	75	74.3	48	71.6	0.71
	Death	26	25.7	19	28.4	

Abbreviation: G1, grade 1 (well differentiation); G2, grade 2 (moderated differentiation); G3, grade 3 (poor differentiation). Special type: includes mucinous adenocarcinoma and signet ring cell adenocarcinoma; pT, pathological primary tumor stage; pN, pathological lymph node stage; TNM, tumor, lymph node, and metastasis stage.

Supplementary Table 4 Clinicopathological features of 47 patients of Image set C from TCGA-COAD.

Variable	Subtype	n	%
Age(y)	≤50	9	0.191
	>50	38	0.809
Gender	Male	21	44.7
	Female	26	55.3
Tumor site	Right colon	23	48.9
	Left colon	19	40.4
Histologic type	G1+G2	38	80.9
	G3+special type	8	17
pT stage	T1+T2	3	6.4
	T3	38	80.9
	T4	6	12.8
pN stage	N1	24	51.1
	N2	23	48.9
TNM stage	IIIA	2	4.3
	IIIB	18	38.3
	IIIC	19	83
DFS status	Non-recurrence	31	66
	Recurrence	16	34
OS status	Alive	19	40.4
	Death	28	59.6

Abbreviation: G1, grade 1 (well differentiation); G2, grade 2 (moderated differentiation); G3, grade 3 (poor differentiation).; Special type: includes mucinous adenocarcinoma and signet ring cell adenocarcinoma; pT, pathological primary tumor stage; pN, pathological lymph node stage; TNM, pathological tumor, lymph node, and metastasis stage.

Supplementary Table 5 Recognition accuracy of the tissue categories of four different convolution neural networks (CNNs) in the Image set A.

CNN model	Top-1 accuracy on ImageNet	Top-5 accuracy on ImageNet	Accuracy on nine classification
VGG19	0.713	0.900	0.975
ResNet50	0.749	0.921	0.969
InceptionV3	0.779	0.937	0.981
InceptionResNetV2	0.803	0.953	0.990

Supplementary Table 6 Predictive performance of nine different machine classifiers in different sets.

	DFS			OS		
Classifier	train	test	validation	train	test	validation
**Five-fold cross validation**						
Decision Tree	0.71	0.78	0.69	0.85	0.76	0.54
Random Forest	0.76	0.74	0.60	0.85	0.76	0.55
Gradient Boosting Decision Tree	0.84	0.78	0.73	0.89	0.83	0.71
AdaBoost Decision Tree	0.73	0.76	0.68	0.80	0.82	0.51
LDA	0.72	0.77	0.61	0.78	0.80	0.54
SVM	0.67	0.78	0.66	0.90	0.76	0.46
Multinomial NB	0.63	0.61	0.59	0.68	0.64	0.54
Bernoulli NB	0.68	0.79	0.60	0.78	0.82	0.43
KNN	0.75	0.65	0.68	0.83	0.76	0.51
**Jackknife test**						
Decision Tree	0.76	0.61	0.70	0.89	0.71	0.55
Random Forest	0.79	0.68	0.56	0.90	0.78	0.58
Gradient Boosting Decision Tree	0.78	0.67	0.69	0.92	0.76	0.61
AdaBoost Decision Tree	0.77	0.68	0.60	0.83	0.76	0.53
LDA	0.74	0.72	0.55	0.80	0.79	0.55
SVM	0.74	0.72	0.64	0.82	0.79	0.53
Multinomial NB	0.65	0.62	0.54	0.74	0.68	0.55
Bernoulli NB	0.69	0.64	0.66	0.81	0.71	0.56
KNN	0.73	0.67	0.64	0.81	0.77	0.49

Abbreviation: LDA, Linear Discriminate Analysis; SVM, Support Vector Machine; NB, Naive Bayes; KNN, K-Nearest Neighbor

Supplementary explanation

The idea of Boosting is to increase the weights of the incorrect samples of last epoch during training so as to enhance the performance of weak classifier. For Gradient Boosting, the negative gradients of last training epoch would be regarded as the criterion for updating the parameters of new training epoch so that the false classified samples could be corrected. The author of Gradient Boosting had proved the aforementioned idea based on the gradient descent of parameter space[1].

The main task of machine learning is to minimize the loss function L(θ), in which θ denotes the parameters of model. The most classical method of optimization is gradient descent, which has the formula of parameter update shown as follows,

$$\theta = \theta - \alpha \frac{\partial}{\partial \theta} L(\theta) \qquad (1)$$

where α is the learning rate.

The Gradient Boosting algorithm utilizes the additive model, which means the previous *m-1*th base classifiers are fixed in the *m*th training epoch,

$$f_m(x) = f_{m\text{-}1}(x) + \rho_m h_m(x) \qquad (2)$$

where $h_m(x)$ denotes the *m*th classifier and ρ_m means the coefficient of base classifier.

Therefore, the aim of the *m*th training process is to minimize the loss function $L(f) = \sum_{i=1}^{N} L(y_i, f_m(x_i))$ so as to obtain the corresponding base classifier. If the $f(x)$ could be regarded as parameters, the gradient descent method could be reused as follows,

$$f_{\mathrm{m}}(x) = f_{m-1}(x) - \rho_m \cdot \frac{\partial}{\partial f_{m-1}(x)} L(y, f_{m-1}(x)) \qquad (3)$$

We could specify the following conclusion from the comparison of formula (2) and (3),

$$h_{\mathrm{m}}(x) \approx -\frac{\partial L(y, f_{m-1}(x))}{\partial f_{m-1}(x)}$$

which means the base classifier $h_m(x)$ could fit the negative gradients of the last training epoch. In other words, it denotes that the L(f) could be optimized through gradient descent. The negative gradients are so called “pseudo residual”. The higher $r = y - f(x)$ presents that the results of classifier $f(x)$ have larger distance from ground truth y, and the errors could be corrected by fit the negative gradients in next training epoch.

References:

1. Friedman, J.H. Greedy function approximation: a gradient boosting machine. *ANN STAT*, 1189-1232 (2001).
